# Association of time-serial changes in ambient particulate matters (PMs) with respiratory emergency cases in Taipei's Wenshan District

**DOI:** 10.1371/journal.pone.0181106

**Published:** 2017-07-21

**Authors:** Jer-Hwa Chang, Shih-Chang Hsu, Kuan-Jen Bai, Shau-Ku Huang, Chin-Wang Hsu

**Affiliations:** 1 Division of Pulmonary Medicine, Department of Internal Medicine, Wan Fang Hospital, Taipei Medical University, Taipei, Taiwan; 2 School of Respiratory Therapy, College of Medicine, Taipei Medical University, Taipei, Taiwan; 3 Emergency Department, Department of Emergency and Critical Medicine, Wan Fang Hospital, Taipei Medical University, Taipei, Taiwan; 4 National Institute of Environmental Health Sciences, National Health Research Institutes, Zhunan, Miaoli County, Taiwan; 5 Lou-Hu Hospital, Shen-Zhen University, Shen-Zhen, China; 6 Research Center for Environmental Medicine, Kaohsiung Medical University, Kaohsiung, Taiwan; 7 Johns Hopkins Asthma and Allergy Center, Johns Hopkins University School of Medicine, Baltimore, Maryland, United States of America; 8 Department of Emergency Medicine, School of Medicine, College of Medicine, Taipei Medical University, Taipei, Taiwan; University of Calcutta, INDIA

## Abstract

Ambient air pollution poses a significant risk for a group of common and often debilitating respiratory diseases, but its direct impact on cause-specific respiratory diseases using emergency room visit (ERV) as an indicator remains to be fully explored. In this study, we conducted a time-series study of ambient PM_2.5_, NO_2_, SO_2_ and their association with ERV for asthma, COPD and pneumonia in a four-year time span. Relative risks for ERV as per log increase in the level of ambient pollutants with time lags of up to 10 days were calculated, using a generalized additive model of Poisson regression. Daily 24-h average concentrations of PM_2.5_ and pollutant gases were obtained from a local Gutting air quality monitoring station. Results showed that the ERVs for pneumonia and asthma were associated with the level of PM_2.5_. The effects of PM2.5 on the risk of ERV for asthma were found to be significant at lag days 1 and 2 with increasing risk of 4.34% [RR: 1.091; CI: 1.020–1.166 (95%)] and 3.58% [RR: 1.074; CI: 1.007–1.146 (95%)], respectively. The ERV for pneumonia was associated with the level of PM_2.5_ at lag days 5, 6 and 7, with increasing risk of 1.92% [RR: 1.039; CI: 1.009–1.070 (95%)], 2.03% [RR: 1.041; CI: 1.009–1.075 (95%)], and 1.82% [RR: 1.037; CI: 1.001–1.075 (95%)], respectively. Further, PM_2.5_, but not NO_2_ and SO_2_, posed a significant risk of ERV for asthma during spring at lag days 0, 1 and 2 (17.12%, RR: 1.408, CI: 1.075–1.238; 15.30%, RR: 1.358 CI: 1.158–1.166; 11.94%, RR: 1.165, CI: 1.004–1.121), which was particularly evident for those who were younger than 75 years of age. In contrast, only PM_2.5_ was a significant risk of ERV for COPD, which was primarily for those who were younger than 75 years of age during summer season at lag days 3, 4 and 5. (26.66%, RR: 1.704, CI: 1.104–2.632; 26.99%; RR: 1.716, CI: 1.151–2.557; 24.09%; RR: 1.619, CI: 1.111–2.360). Collectively, these results suggested significant seasonal variation and differential time lag effects of PM_2.5_ on ERV for asthma, COPD and pneumonia.

## Introduction

Ambient air pollution is associated with respiratory diseases and, in some cases, mortality [1–6], wherein exposure to ambient PMs poses a significant risk for asthma, COPD, pneumonia and cancer[7–12]. In fact, the WHO has reported that ambient air pollution is responsible for 3.7 million deaths in 2012, representing 6.7% of total deaths worldwide, raising significant environmental, public health, medical and economic concerns[13,14]. These documented impacts of ambient air pollution also highlight an urgent need for better understanding of the nature of the disease-causing pollutants and their exact impact on the occurrence of diseases, particularly for a group of common and often debilitating respiratory diseases.

While several studies have demonstrated the effects of PM_2.5_ on the number of hospital admissions for respiratory diseases[15–17], few reports have directly investigated the effects of PM_2.5_ on cause-specific respiratory diseases using ERVs as an indicator. Most of the epidemiologic and exposure studies on health effects have primarily focused on disease association, and while the time series and case-crossover studies exploring the delayed (or ‘lagged’) association between exposure and outcome have been informative, comparative analysis of the delayed associations among different diseases has been scarce. The objective of this study was thus to evaluate the time-lag and seasonal effects of air pollutants, including PM_2.5_, on the incidence of emergency cases for respiratory diseases over a four-year time frame in a metropolitan medical center, where the primary patient population came from the same geographical location. Herein, we report significant seasonal variation and differential time lag effects among three common respiratory diseases, including pneumonia, chronic obstructive pulmonary disease and asthma.

## Materials and methods

### Health data

The data on daily emergency department visit for respiratory disease were obtained from Wan Fang Medical Center, Taipei, Taiwan, during the period of 2012 to 2015 (1461 days). The hospital is affiliated with Taipei Medical University and is located within the metropolitan area of Taipei City, Taiwan, at Wenshan District, and has had more than 65,000 emergency visits annually. Relevant data elements included a unique patient identifier number, admission date, admission source, primary and secondary International Classification of Diseases, 9th Revision (ICD-9) diagnosis codes. In the study, we considered information on emergency hospital admissions for respiratory diseases, including visits for pneumonia (480–486), chronic obstructive pulmonary disease (491, 492, 496), and asthma (493).

### Environmental data

Daily 24-h average concentrations of PM_2.5_, nitrogen dioxide (NO_2_) and sulfur dioxide (SO_2_) were obtained from the Environmental Protection Administration’s (EPA) Taiwan Air Quality Monitoring Network (TAQMN). The hourly air pollution data collected from Gutting air quality monitoring station with distance of 4 km to Wan Fang Medical Center were utilized. The 24 h average levels of the pollutants were computed. Daily information on mean temperature was provided by the Taipei Observatory of the Central Weather Bureau.

### Statistical analyses

Descriptive statistics and correlation patterns between air pollutants, and meteorological factors were analyzed. The Distributed-lag model analysis was performed to study the association between the number of emergency visits and the level of pollutants at different lags, controlling for possible confounding factors, such as mean daily temperature, NO_2_ and SO_2_. Relative risk of emergency visit was calculated using a generalized additive model based on Poisson distribution allowing for over-dispersion (quasi-likelihood). For the independent variables, a natural cubic spline with 6 degrees of freedom per year was used to account for seasonal variability. Regarding the concentration of PM_2.5_, a basis function was used for the lagged variable, using a polynomial of the third degree (df = 4), assuming that the relationship between the dependent variable and the predictor was linear. We also performed separate analyses of seasonal variations, including those parameters in the spring (March-May), summer (June-August), fall (September-November) and winter (December-February) periods. All findings were presented as relative risks (RRs) of emergency visit with 95% confidence intervals (95% CI). The reference value for PM_2.5_ concentrations was set at 10 μg/m^3^. Relative risks are shown as per log-increase in PM concentrations from the reference. All analyses were conducted in R 3.2.4 and the DLNM[18] package were used.

### Ethics approval

For this retrospective observational study, our IRB waived the need for consent. Also, the data was accessed anonymously. Ethics approval was granted by the Ethics Committee of Taipei Medical University—Joint Institutional Review Board. (TMU-JIRB NO: N201611011)

## Results

During the study period of a 4-year time span between 2012–2015, the total number of ERVs for respiratory diseases were 5335, in which pneumonia represented the majority (69.9%) of ERV, while ERV for two other respiratory diseases, asthma and COPD, were less prominent (Table 1). Also, there were, on average 3.65 ERVs per day for respiratory diseases, with daily means of 0.58 0.55, 2.55 and 0.55 ERV for asthma, pneumonia and COPD, respectively.

**Table 1:** Daily frequency of hospital emergency visits.

	Number of Visit	Daily Mean
Total	5335	3.65
Asthma	800	0.55
Pneumonia	3729	2.55
COPD	806	0.55

**Fig 1:**
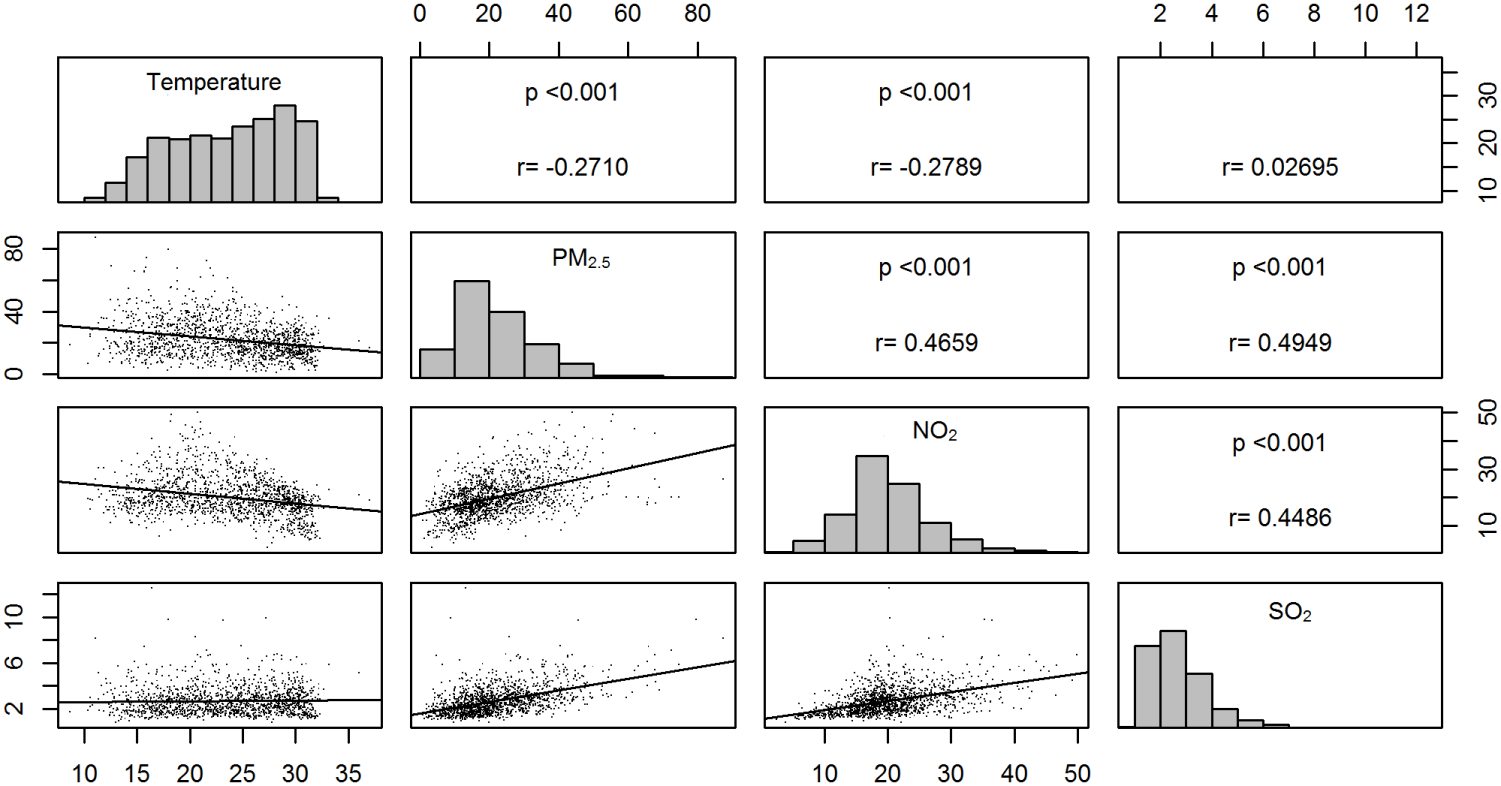
Correlation matrix for ambient air pollutants in Taipei, Taiwan, 2012–2015; Temperature: °C, PM2.5: ture: aipei,ppb, SO2: ppb.

Based on the dataset of a stationary monitoring station within the vicinity of Wan-Fang Medical Center, the mean 24-h PM_2.5_ concentration was 21.96 μg/m^3^, ranging from 1.00 to 87.33 μg/m^3^. Higher particle mass concentrations were typically observed during winter. The descriptive statistics for the corresponding environmental data are shown in Table 2. Fig 1 depicts the Pearson correlation matrix for the selected variables. Results showed that the concentrations of PM_2.5_ were positively correlated with the concentrations of SO_2_ and NO_2_, with both p values less than 2.2X10^-16^, and that the pollutant parameters were significantly correlated to each other (p < 0.001).

**Table 2:** Distribution of daily temperature, and air pollution variables in Taipei, Taiwan, 2012–2015.

		Min	Max	Mean	Median	SD
**Year**	Temperature (°C)	8.63	37.00	23.55	24.21	5.44
	PM2.5 (μg/m^3^)	1.00	87.33	21.96	19.71	11.60
	NO2 (ppb)	2.38	49.88	20.14	19.36	6.73
	SO2 (ppb)	0.82	12.53	2.70	2.47	1.18
**Spring**	Temperature (°C)	12.71	31.08	22.51	22.96	4.16
	PM2.5 (μg/m^3^)	3.83	63.04	26.11	24.60	11.36
	NO2 (ppb)	9.83	49.25	23.55	22.70	7.13
	SO2 (ppb)	0.82	9.73	2.86	2.73	1.18
**Summer**	Temperature (°C)	24.88	37.00	29.52	29.75	1.68
	PM2.5 (μg/m^3^)	1.92	44.00	17.14	16.25	7.72
	NO2 (ppb)	2.38	32.04	17.34	17.95	5.20
	SO2 (ppb)	1.08	6.62	2.71	2.64	0.98
**Fall**	Temperature (°C)	13.50	32.13	24.89	24.77	3.40
	PM2.5 (μg/m^3^)	1.00	54.38	19.23	17.77	10.00
	NO2 (ppb)	3.82	45.58	17.39	17.18	5.03
	SO2 (ppb)	0.83	9.88	2.41	2.14	1.07
**Winter**	Temperature (°C)	8.63	23.71	17.15	17.13	2.74
	PM2.5 (μg/m^3^)	2.00	87.33	25.41	22.92	13.85
	NO2 (ppb)	6.41	49.88	22.29	20.78	6.81
	SO2 (ppb)	0.95	12.53	2.83	2.51	1.41

**Fig 2:**
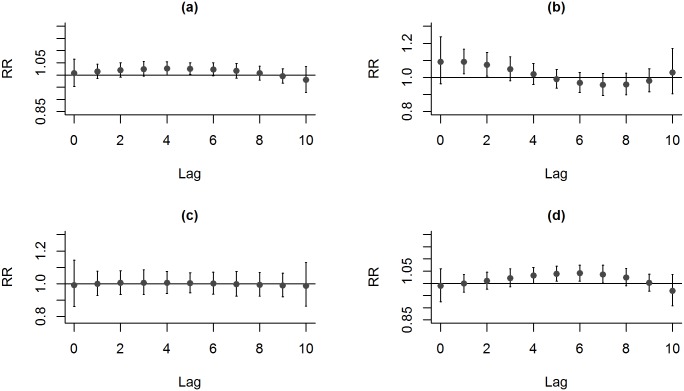
Lag-response curve for every 10 μg/m^3^ increase of PM_2.5_ for respiratory diseases. (a) all three respiratory diseases, (b) asthma, (c) COPD, and (d) pneumonia.

For the Distributed-lag model analysis, based on Poisson regression, with the three pollutants analyzed together, adjusted for temperature, we obtained their relative risks and their respective confidence intervals of 95% for ERV. The effects of PM2.5 on the risk of ERV for total respiratory diseases were found to be significant at lag day 5 with increasing risk of 1.27% [RR: 1.026; CI: 1.001–1.051 (95%)] (Fig 2a). The PM_2.5_ levels and ERVs for all three diseases demonstrated that ERVs for pneumonia and asthma were associated with the level of PM_2.5_ (Fig 2b and 2d). The effects of PM_2.5_ on the risk of ERV for asthma were found to be significant at lag days 1 and 2 (Fig 2b). At lag day 1 and 2, the risk of ERVs for asthma increased by 4.34% [RR: 1.091; CI: 1.020–1.166 (95%)] and 3.58% [RR: 1.074; CI: 1.007–1.146 (95%)], respectively, as per 10 μg/m3 increase in PM_2.5._ Also, the effects of PM_2.5_ on the risk of ERV for pneumonia were found to be significant at lag days 5, 6 and 7 (Fig 2d). At lag day 5, 6 and 7, the risk of ERVs for pneumonia increased by 1.92% [RR: 1.039; CI: 1.009–1.070 (95%)], 2.03% [RR: 1.041; CI: 1.009–1.075 (95%)], and 1.82% [RR: 1.037; CI: 1.001–1.075 (95%)], respectively, as per 10 μg/m^3^ increase in PM_2.5_. In contrast, no significant association of ERVs was noted for any one of the three respiratory diseases with the levels of NO_2_ and SO_2_ (S1 Fig).

**Fig 3:**
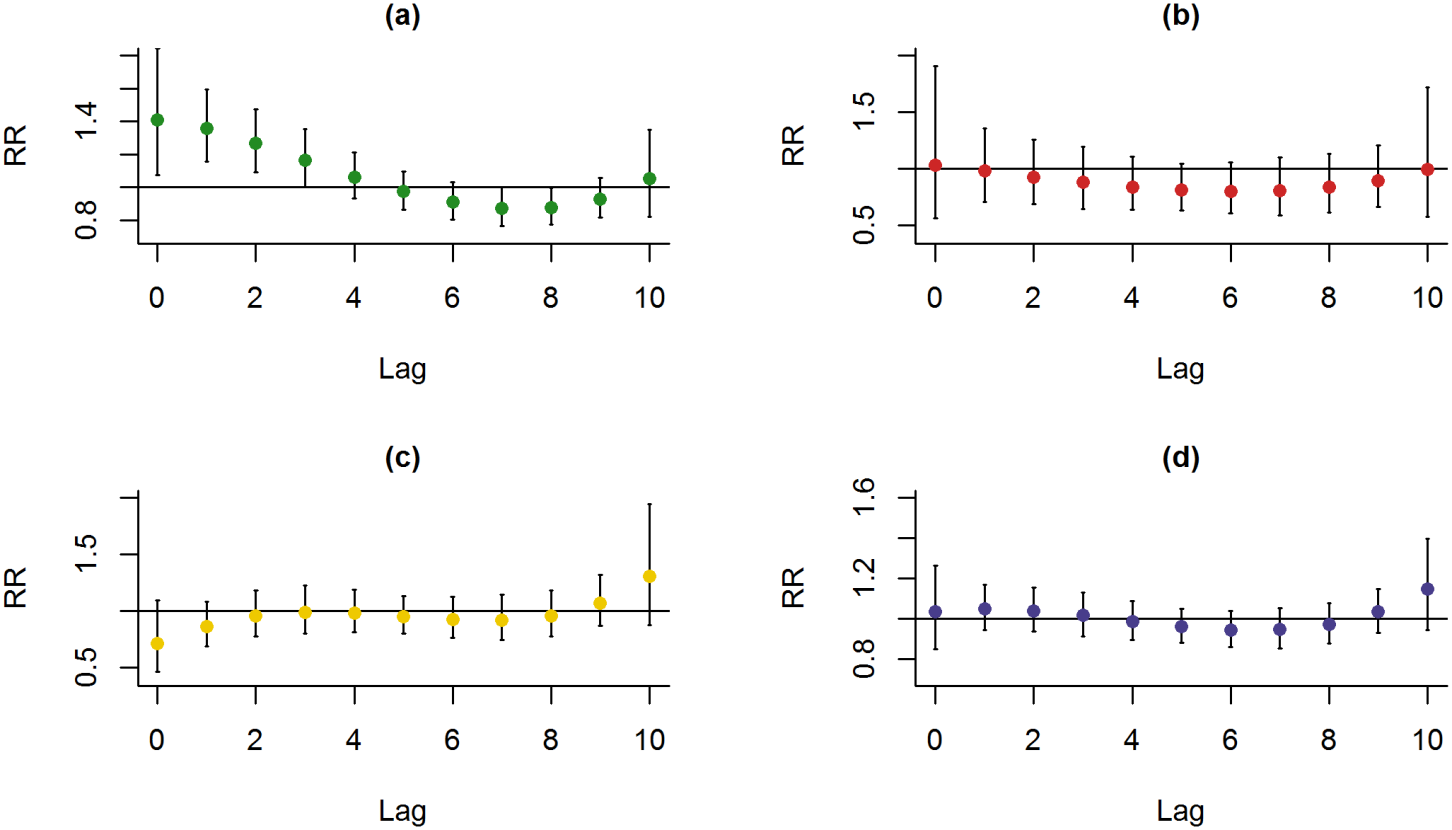
Relative risks of ERVs for asthma with an increase of 10 μg/m^3^ in PM_2.5_ in different seasons. (a) Spring, (b) Summer, (c) Fall and (d) Winter.

When examining ERV by different seasons, an increase in PM_2.5_ concentration was associated with significant increase in ERV for asthma and COPD. In the context of asthma, a total of 800 asthma visits were analyzed, of whom 13.39% were admitted. During the spring, significantly lower frequency of admission was found as compared with those during the fall and winter. Significantly, increased risk of ERVs for asthma was noted during spring at lag days 0, 1 and 2 (17.12%, RR: 1.408, CI: 1.075–1.238; 15.30%, RR: 1.358 CI: 1.158–1.166; 11.94%, RR: 1.165, CI: 1.004–1.121; Fig 3). Interestingly, no significant association of ERVs was found for asthma during spring with the levels of NO_2_ or SO_2_ (S2 Fig).

**Fig 4:**
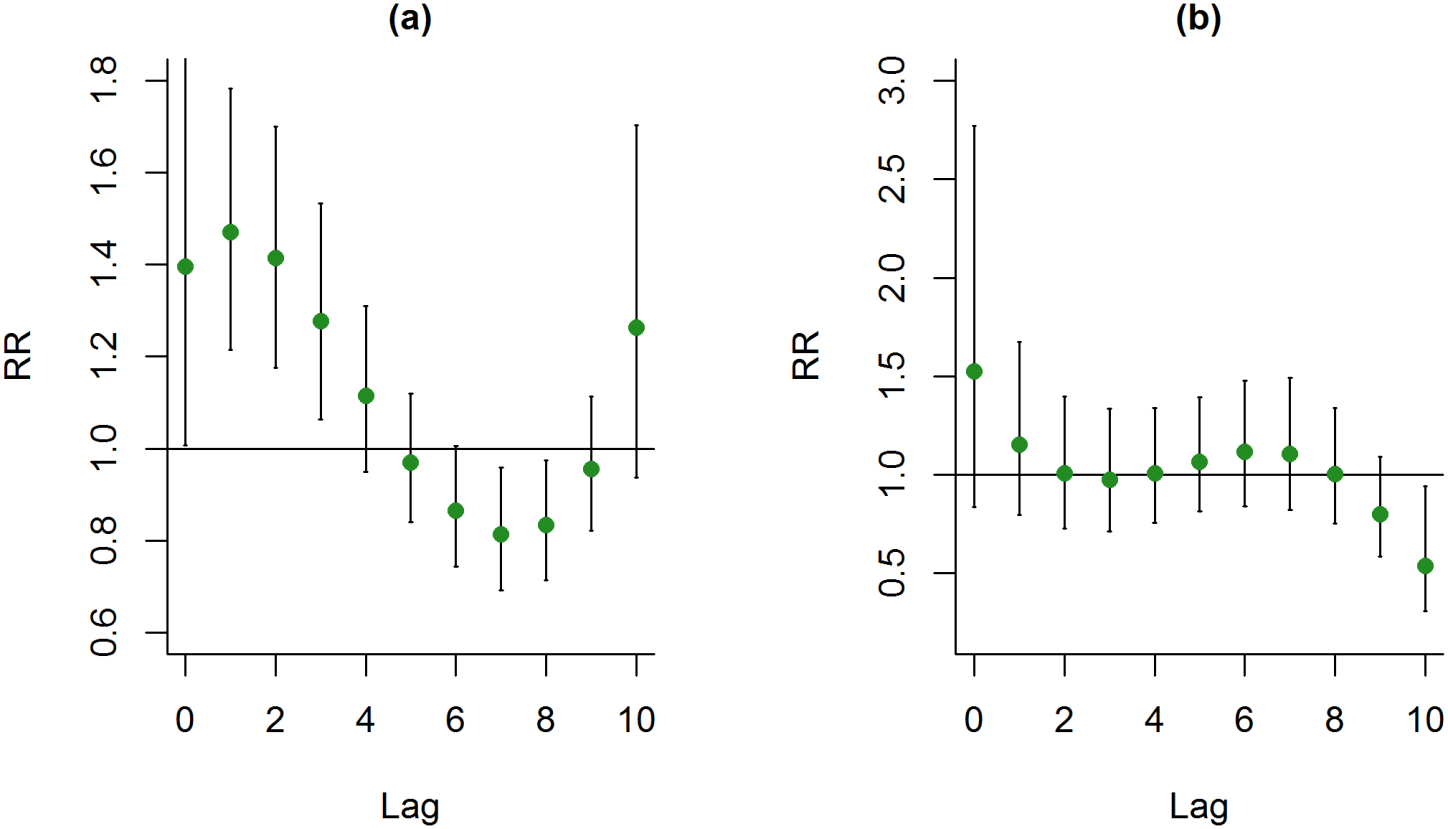
Lag-response curve for every 10 μg/m^3^ increase of PM_2.5_ for asthmatic patients stratified by age. (a) Asthmatic patients who were < 75 years old and (b) Asthmatic patients who were 75 years of age or older.

When the dataset was stratified by age, it was noted that the patients with asthma who were older than 75 years had significant higher admission rate, and for asthma patients of younger than 75 years of age, a 10 μg/m^3^ increase in PM_2.5_ during spring was significantly associated with ERV at lag days 0, 1, 2 and 3 (16.69%, RR: 1.396, CI: 1.007–1.936; 19.31%, RR: 1.471, CI: 1.214–1.783; 17.33%, RR: 1.414, CI: 1.176–1.701; 12.22%, RR: 1.277, CI: 1.063–1.534; Fig 4a). PM_2.5_ appeared to show no significant effect on asthma patients of older than 75 years of age during spring (Fig 4b).

**Fig 5:**
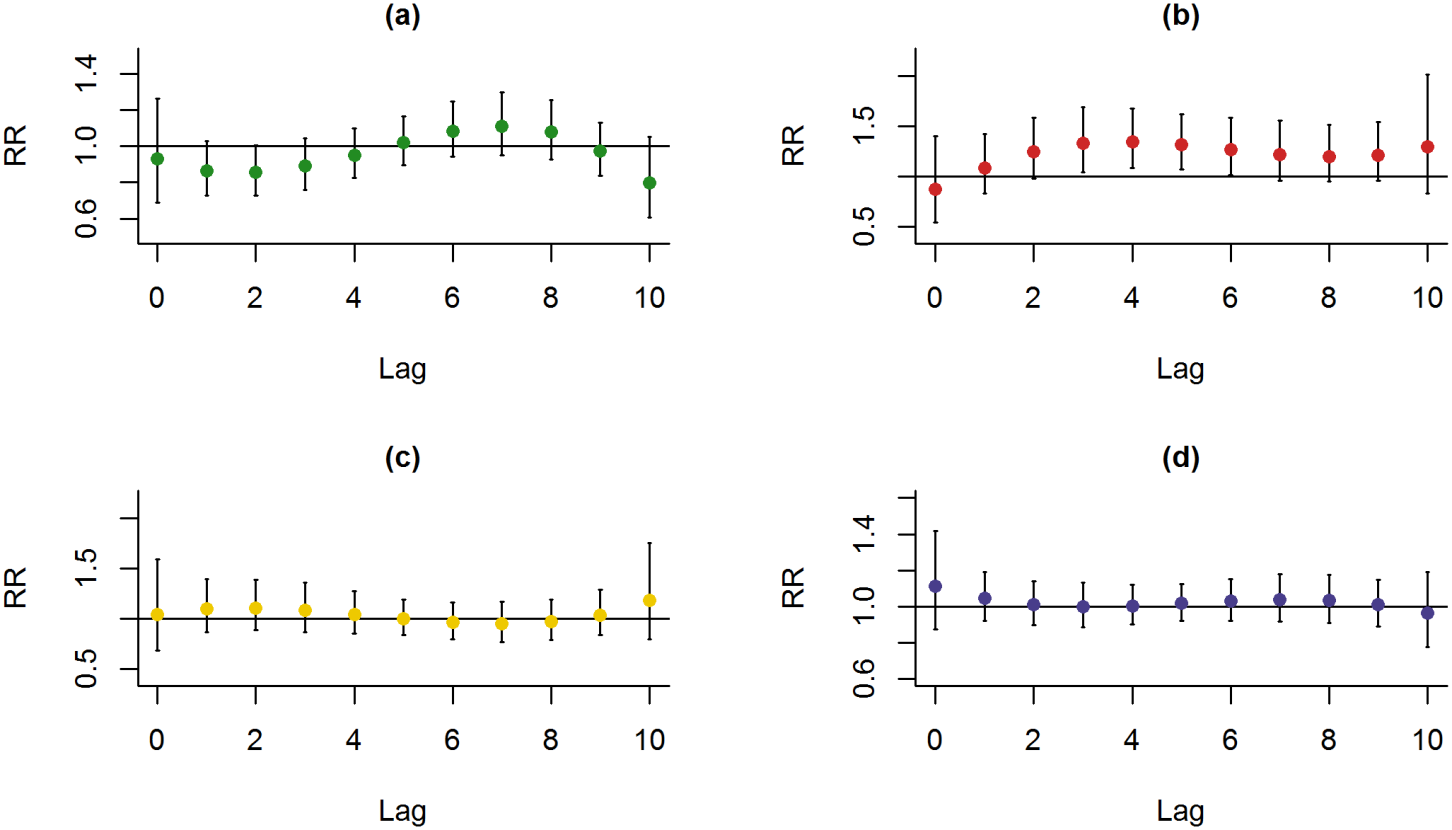
Relative risks of ERVs for COPD with an increase of 10 μg/m³ in PM_2.5_ at different seasons. (a) Spring, (b) Summer, (c) Fall and (d) Winter.

As for COPD, a total of 806 COPD visits were analyzed, of whom 40.81% were admitted, and no significant difference was seen for the frequency of admission among different seasons. During summer, significant associations at lag days 3, 4, 5 and 6 were found between PM_2.5_ and ERV for COPD (14.19%, RR: 1.328, CI: 1.045–1.688; 14.86%; RR: 1.346, CI: 1.082–1.676; 13.81%; RR: 1.318, CI: 1.072–1.621; 11.89%; RR: 1.269, CI: 1.04614–1.587, respectively; Fig 5). During summer, there was no significant association between the level of NO_2_ and ERV for COPD; also, SO_2_ did not appear to be a risk factor (S3 Fig).

**Fig 6:**
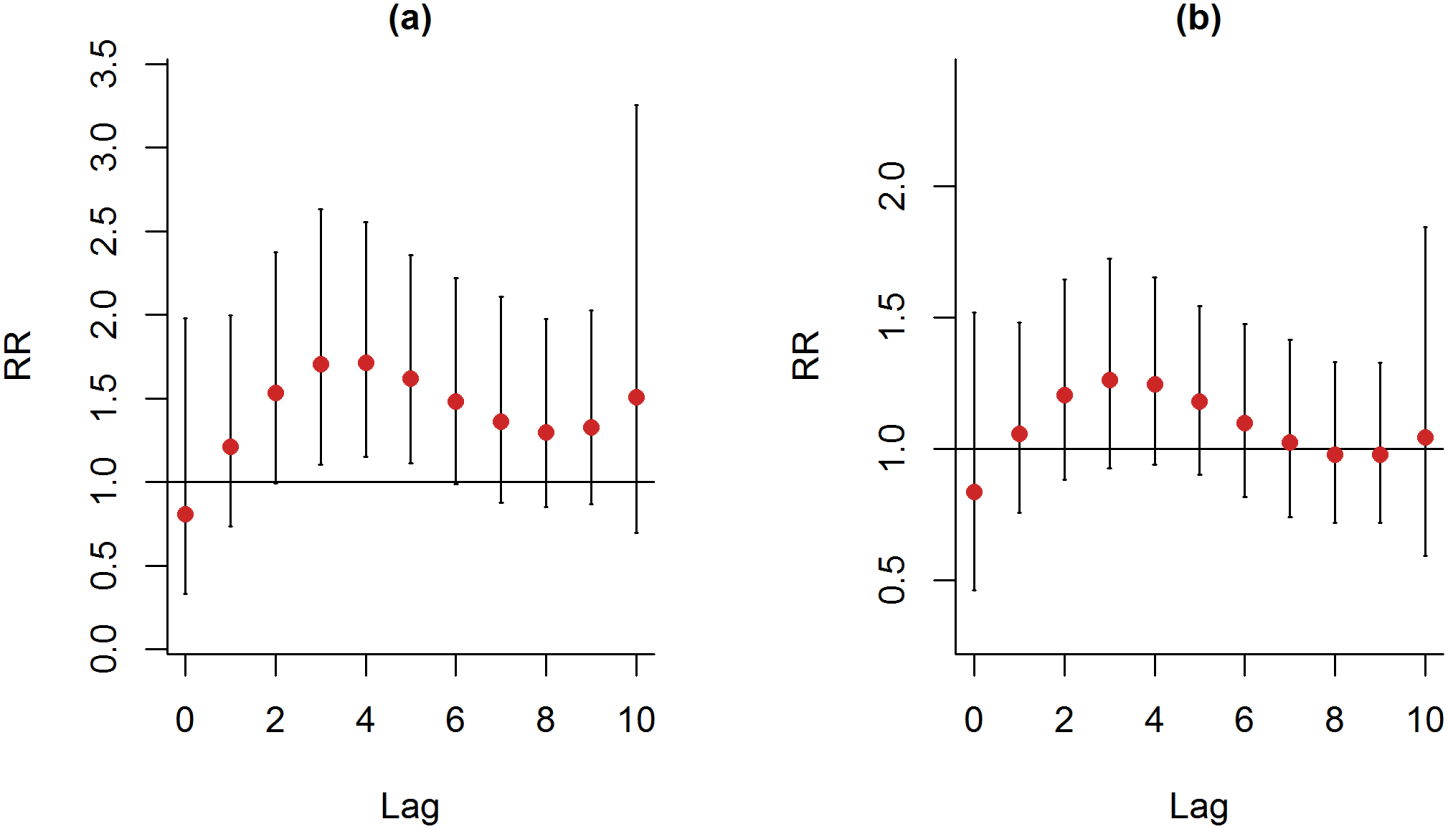
Lag-response curve for every 10 μg/m^3^ increase in PM_2.5_ for patients with COPD in different age groups. (a) COPD patients who were < 75 years old and (b) COPD patients who were 75 years of age or older.

The patients with COPD were also divided into two age groups: ≥ 75 years of age and<75 years of age. More than half of the patients with COPD who visited the emergency department were older than 75 years of age. By comparison with winter, during summer, a higher percentage of COPD patients who were older than 75 years visited the emergency department. Also, the patients with COPD who were older than 75 years had significant higher admission rate. For COPD patients of younger than 75 years of age, a 10 μg/m^3^ increase in PM_2.5_, during summer, was significantly associated with ERV at lag days 3, 4 and 5. (26.66%, RR: 1.704, CI: 1.104–2.632; 26.99%; RR: 1.716, CI: 1.151–2.557; 24.09%; RR: 1.619, CI: 1.111–2.360; Fig 6a). PM_2.5_ didn’t show any significant effect on COPD patients of older than 75 years of age during summer (Fig 6b).

## Discussion

In this study, we attempted to identify the relationship between the level of ambient air pollution, including the level of PM_2.5_, and ERV for three different respiratory diseases in a four-year time series study. While most of previous epidemiologic studies used number of hospital admissions as an indicator as an acute exacerbation indicator, the number of ERVs would be more relevant to disease exacerbation and would be more pertinent to investigate the time lag effects. Our study showed significant seasonal variation and differential time lag effects on ERV for asthma, COPD and pneumonia. In this study, our findings reveal associations between PM_2.5_ and ERVs for asthma and pneumonia. However, when the analyses were separated by season, increased risks of ERV for asthma and COPD with increasing ambient PM_2.5_ levels were noted in the spring and in the summer, respectively.

Several studies have demonstrated the effects of PM_2.5_ on the number of hospital admissions due to pneumonia, COPD and asthma [8,19,20]. Nevertheless, few studies have investigated the effects of PM_2.5_ on cause-specific respiratory diseases using ERV as a morbidity indicator. Most studies, which used ERV as a morbidity indicator, were conducted in western developed countries and there is still a need to assess the health effects of PM_2.5_ exposure in Asia countries. Further, this study is in accordance with previous findings of adverse effects of PM_2.5_ on asthma, COPD, and pneumonia admissions on both warm and cool days in Taipei [8,19,20]. However, our data only revealed the significant association during the specific season. In this study, the PM_2.5_ level was found to be associated with asthma ERV during spring, COPD ERV during summer and pneumonia during all 4 seasons. Interestingly, higher particle matter concentrations were observed during winter. This seasonal difference has also been reported in Kaohsiung, Taiwan. The prior work by Hwanh et al. [21] assessed the influence of PM_2.5_ on hospital admissions for COPD in Kaohsiung, and observed an increased risk of COPD admissions associated with ambient PM_2.5_ levels on cool days. The difference in the effect of PM_2.5_ on ERV for cause-specific respiratory diseases in different seasons and location might be due to the variations in PM_2.5_ constituents. PM is formed by different compounds. Depending on sources, PM composition can be different among cities [22] and shows obviously seasonal patterns, and some components of PM also show seasonal patterns. [23–25].

In this study, PM_2.5_ showed different lag effect on different diseases. The level of PM_2.5_ was associated with asthma exacerbation ERV at lag 0 day, while it had an impact on COPD ERV at the lag 3 day. Moreover, the pneumonia ERV also showed an association with the level of PM_2.5_ at the lag 5 day. The different lag effect suggests that the different underlying mechanism of the diseases can be elicited by PM_2.5_. The mechanisms of the adverse effects of PM_2.5_ on the respiratory system have been suggested including: oxidative stress and altered immunity. [26] Several studies have suggested that PM_2.5_ can induce acute oxidative stress and inflammation in respiratory system. [27–29] A resent research demonstrated that the pulmonary inflammation and oxidative stress pathway is the first to respond to PM_2.5_ exposure. [6] Moreover, exposure of PM_2.5_ also can suppress phagocytosis of bacteria [30,31] and enhance pneumococcal adhesion to epithelial cells. [32] Also, the inhaled PM can induce direct oxidative stress and changes to growing conditions of respiratory microbiome. Disrupting the community structure of the microbiome could then result in downstream respiratory tract infection.[33] In our study, the increased level of PM_2.5_ was associated with asthma exacerbation at early lag day. The results suggest that, in asthma exacerbation, the PM_2.5_ may function as a trigger to directly induce asthma attack. In terms of AECOPD and pneumonia ERV, the positive associations with PM_2.5_ concentrations were at later lag day. The data suggests that PM_2.5_ might have a priming effect on respiratory tract and precondition the lung for further infection or inflammatory triggers.

For age subgroup analysis, a clear positive association between PM and respiratory disease was not evident overall in our study. There was an increase in AECOPD ERVs in patients who were younger than 75 years, with increase in PM_2.5_ in the summer. Likewise, for the <75 years of age group, ERV for asthma exacerbation were positively associated with PM_2.5_ during the spring. People who are younger tend to spend more time outdoor, which might explain the positive effects of PM_2.5_ exposures in younger patients.

Several limitations of the current study are clearly evident. This includes the possibility of measurement error, an inherent limitation of epidemiology studies using air pollution measurements from fixed air monitoring sites. Previous research has raised a question about using stationary PM data might lead to diminishing the accuracy of exposure-response estimates compared to personal exposures. [34] In addition, by comparing with other studies, our population size of the study area was relatively small. However, the problem could be solved by using a longer study period. A pervious study demonstrated that the power of a time-series study of the acute health effects of air pollution can be increased by increasing either the mean daily count of the outcome or the time-series length. [35] Our current data suggested that the composition of PM_2.5_ might play a stronger role in disease pathophysiology than the concentration of PM. Also, PM might influence the disease via different mechanisms and cause different lag effects. The majority of the epidemiology studies used PMs as air pollution indicators and combined the data of multiple cities without analyzing the composition of PM. This type of study design might blunt the sensitivity of epidemiologic studies for detecting effects of air pollution on the respiratory disease. In conclusion, our study provides evidence that PM_2.5_ can increase the risk of respiratory ERVs, specifically for asthma, AECOPD and pneumonia.

## Supporting information

S1 Fig Lag-response curve for various diseases as indicated, with a 10-unit increase of NO_2_ (panel a through c) and SO_2_ (panel d to f). (PPTX)

S2 Fig Relative risks of ERVs for asthma with an increase of (a) 10 ppm of NO_2_ and (b) of 1 ppb of SO_2_ during the Spring season. (PPTX)

S3 Fig Relative risks of ERVs for COPD with an increase of (a) 10 ppm of NO_2_ and (b) of 1 ppb of SO2 during the Summer season. (PPTX)

S1 File The data set underlying the findings in our study in the manuscript. (TXT)

## Abbreviations

COPD: Chronic obstructive pulmonary disease
ERV: Emergency room visit
PM: Particulate matter
WHO: World Health Organization
