# Supplementary material for: Association of time-serial changes in ambient particulate matters (PMs) with respiratory emergency cases in Taipei's Wenshan District
Source: PLoS One. 2017 Jul 21;12(7):e0181106. doi: 10.1371/journal.pone.0181106 (PMC5521777; doi:10.1371/journal.pone.0181106)
Supplement: S1 Fig — (PPTX) [file pone.0181106.s001.pptx]

## Slide 1
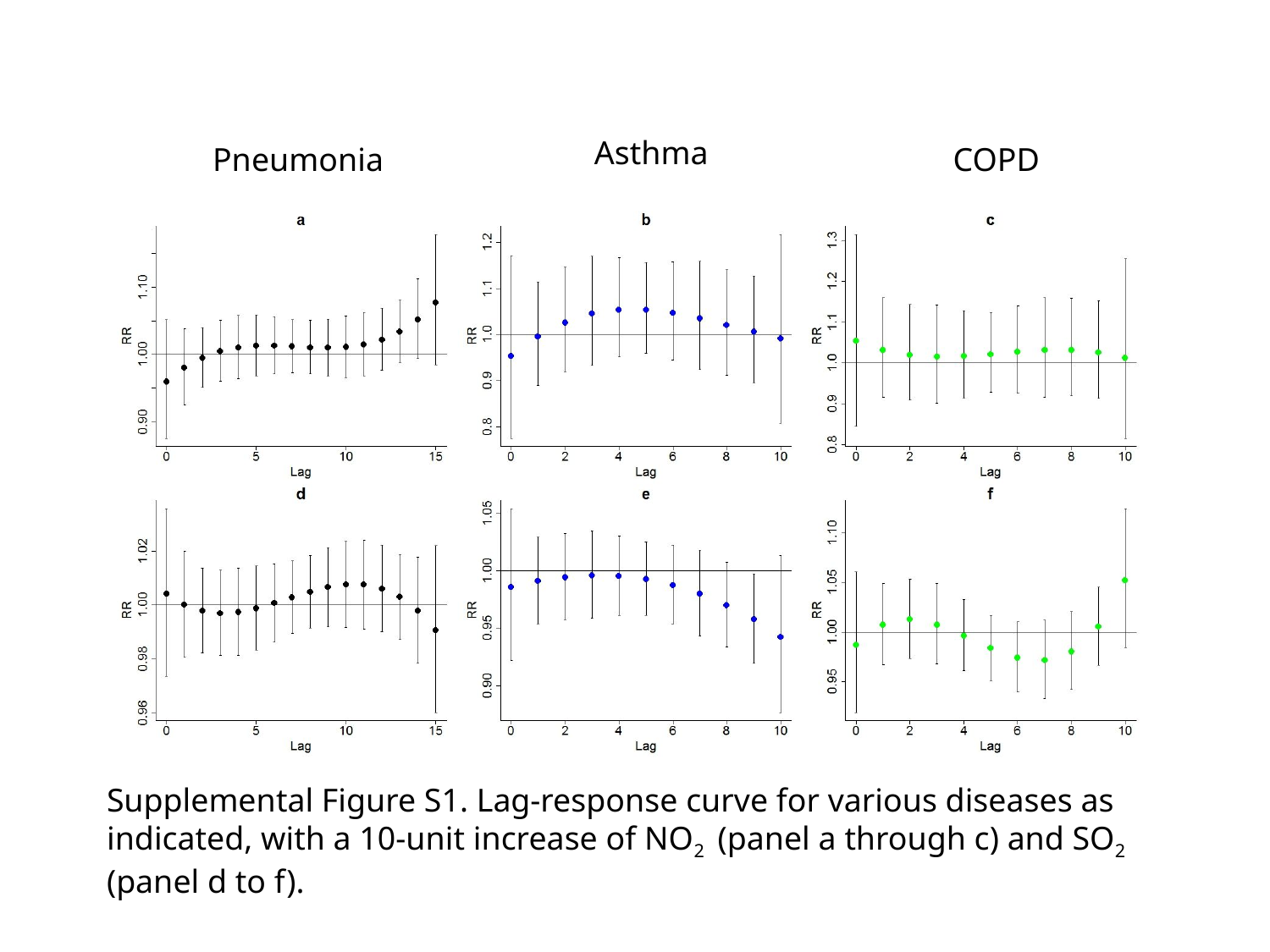

Asthma
Pneumonia
COPD
Supplemental Figure S1. Lag-response curve for various diseases as indicated, with a 10-unit increase of NO2 (panel a through c) and SO2 (panel d to f).
