# Supplementary material for: Association of time-serial changes in ambient particulate matters (PMs) with respiratory emergency cases in Taipei's Wenshan District
Source: PLoS One. 2017 Jul 21;12(7):e0181106. doi: 10.1371/journal.pone.0181106 (PMC5521777; doi:10.1371/journal.pone.0181106)
Supplement: S3 Fig — (PPTX) [file pone.0181106.s003.pptx]

## Slide 1
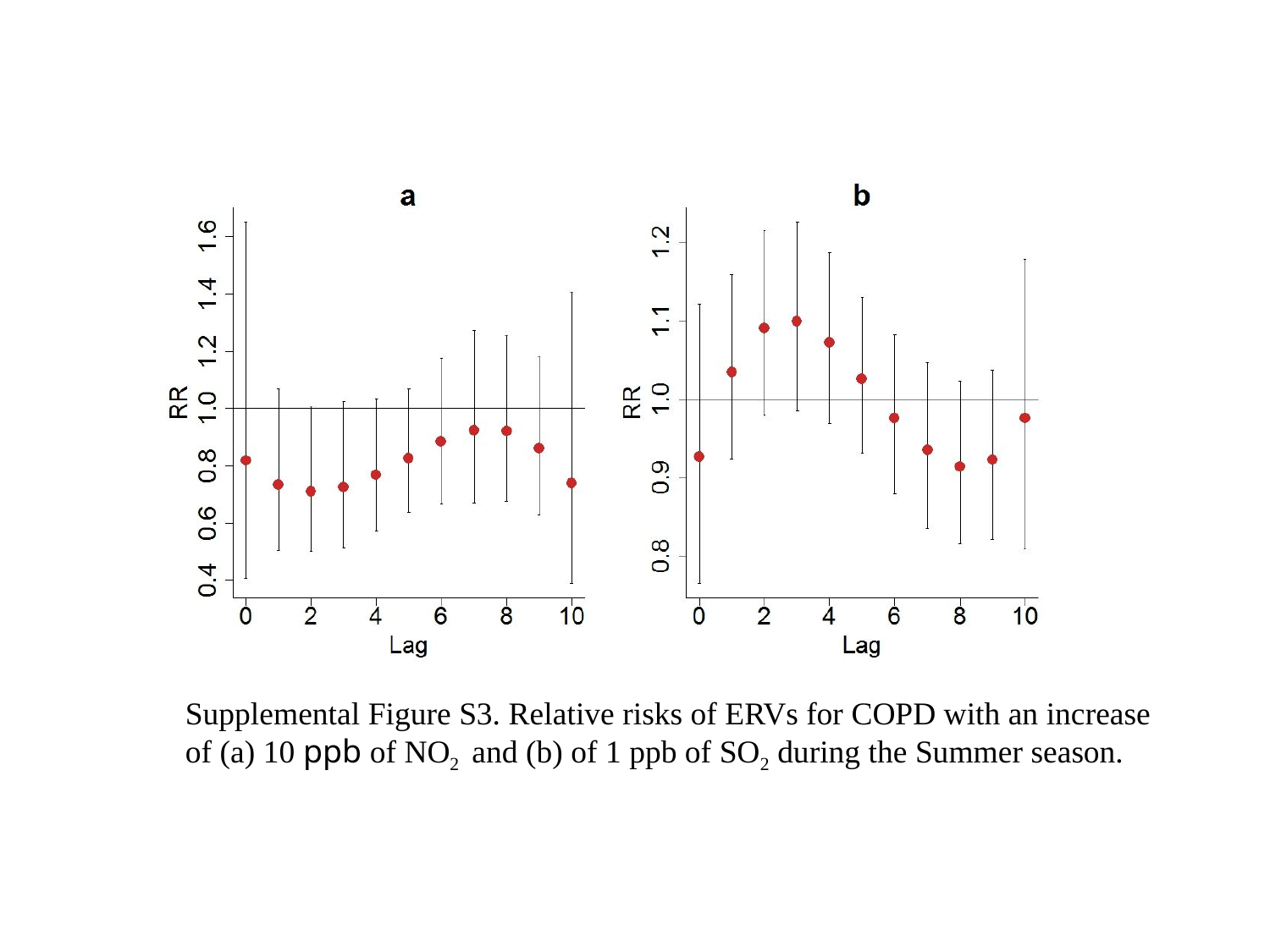

Supplemental Figure S3. Relative risks of ERVs for COPD with an increase of (a) 10 ppb of NO2 and (b) of 1 ppb of SO2 during the Summer season.
